# Chromosomal reference genome sequences for the malaria mosquito, *Anopheles coustani*, Laveran, 1900

**DOI:** 10.12688/wellcomeopenres.22983.1

**Published:** 2024-09-26

**Authors:** Lemonde B. A. Bouafou, Diego Ayala, Boris K. Makanga, Nil Rahola, Harriet F. Johnson, Haynes Heaton, Martin G. Wagah, Joanna C. Collins, Ksenia Krasheninnikova, Sarah E. Pelan, Damon-Lee B. Pointon, Ying Sims, James W. Torrance, Alan Tracey, Marcela Uliano-Silva, Jonathan M.D. Wood, Katharina von Wyschetzki, Scientific Operations: DNA Pipelines collective, Shane A. McCarthy, Daniel E. Neafsey, Alex Makunin, Mara K N Lawniczak

**Affiliations:** 1 MIVEGEC, Univ. Montpellier, CNRS, IRD, Montpellier, France; 2 ESV, Centre Interdisciplinaire de Recherches Médicales de Franceville (CIRMF), Franceville, Gabon; 3 Medical Entomology Unit, Institut Pasteur de Madagascar, Antananarivo, Madagascar; 4 Département de Biologie et Écologie Animale, Institut de Recherche en Écologie Tropicale, Libreville, Gabon; 5 Scientific Operations, Wellcome Sanger Institute, Hinxton, England, UK; 6 CSSE, Auburn University, Auburn, Alabama, USA; 7 Tree of Life, Wellcome Sanger Institute, Hinxton, England, UK; 8 Department of Genetics, University of Cambridge, Cambridge, England, UK; 9 Department of Immunology and Infectious Diseases, Harvard T.H. Chan School of Public Health, Boston, MA, USA; 10 Infectious Disease and Microbiome Program, Broad Institute, Cambridge, MA, USA

**Keywords:** Anopheles coustani, African malaria mosquito, genome sequence, chromosomal

## Abstract

We present genome assembly from individual female *An. coustani* (African malaria mosquito; Arthropoda; Insecta; Diptera; Culicidae) from Lopé, Gabon. The genome sequence is 270 megabases in span. Most of the assembly is scaffolded into three chromosomal pseudomolecules with the X sex chromosome assembled for both species. The complete mitochondrial genome was also assembled and is 15.4 kilobases in length.

## Species taxonomy

Animalia; Arthropoda; Insecta; Diptera; Culicidae; Anophelinae; Anopheles; *Anopheles coustani*; Laveran, 1900 (NCBI txid:139045).

## Background

*Anopheles coustani* (Laveran, 1900) belongs to the Coustani group together with the morphologically similar species *An. crypticus*, *An. fuscicolor*, *An. namibiensis*, *An. paludis*, *An. symesi*, *An. tenebrosus*, *An. caliginosus* and *An. ziemanni* ^1^. Although this mosquito was first described from Madagascar ^2^, it is widespread throughout the African continent. The larvae of *An. coustani* prefer to breed in natural clear water with aquatic vegetation while adults typically rest and feed outdoors ^3, 4^. The feeding preference of *An. coustani* is primarily zoophilic, including wild ungulates, but this zoophilic tendency greatly varies at a local scale from opportunistic to anthropophilic behaviour ^4– 7^. Regarding malaria transmission, *An. coustani* is considered a secondary vector, leading to the species being understudied. However, its epidemiological role in malaria transmission varies from minor importance to locally major vector, as in Madagascar ^8^. The species has been found infected with various human *Plasmodium* species including *P. falciparum*, *P. vivax* and *P. malariae* ^5, 9, 10^. In Madagascar and Cameroon, *An. coustani* was suspected to significantly contribute to malaria outbreaks and sustain malaria transmission ^8, 10^. Apart from human *Plasmodium* species, *An. coustani* has been involved in the transmission of other Haemosporidian parasites (including Hepatocystis) and a variety of arboviruses, including Rift Valley fever and Zika virus ^11– 13^.

Early genetic works enabled distinguishing this species from its sister species, *An. crypticus*. This distinction was based mainly on a fixed chromosomal inversion of the X chromosome ^14^. Very few studies have focused on the genetics of *An. coustani*, for example ^15^ analysed the genetic diversity of *An. coustani*, using COI and ITS2 markers in 50 samples from several locations across Africa. The authors highlighted the existence of two genetic groups with a structure that was not geographically dependent. However, the authors could not rule out the possibility that *An. coustani* and *An. crypticus* are two separate species. One of the most important genomic studies carried out on *An. coustani* is the publication of its complete mitogenome, making available an interesting resource for phylogenetic analyses based on mitochondrial DNA ^16^. Nonetheless, research on the nuclear DNA sequence is currently lacking and will be greatly facilitated by this new chromosomal reference genome.

The genome of the African malaria mosquito, *Anopheles coustani*, was sequenced as part of the Anopheles Reference Genomes Project (PRJEB51690). Here we present a chromosomally complete genome sequence for *Anopheles coustani*, based on a single wild-caught female.

## Genome sequence report

The genome was sequenced from a single female *Anopheles coustani* caught in Lopé, Gabon (-0.143, 11.610) in April 2019 ^17^. A total of 33-fold coverage in Pacific Biosciences single-molecule HiFi long reads (N50 11.273 kb) and 78-fold coverage in 10X Genomics read clouds were generated. Primary assembly contigs were scaffolded with chromosome conformation Hi-C data from an unrelated female individual. Manual assembly curation corrected 3 missing joins or misjoins, reducing the scaffold number by 0.7%.

**Figure 1.**
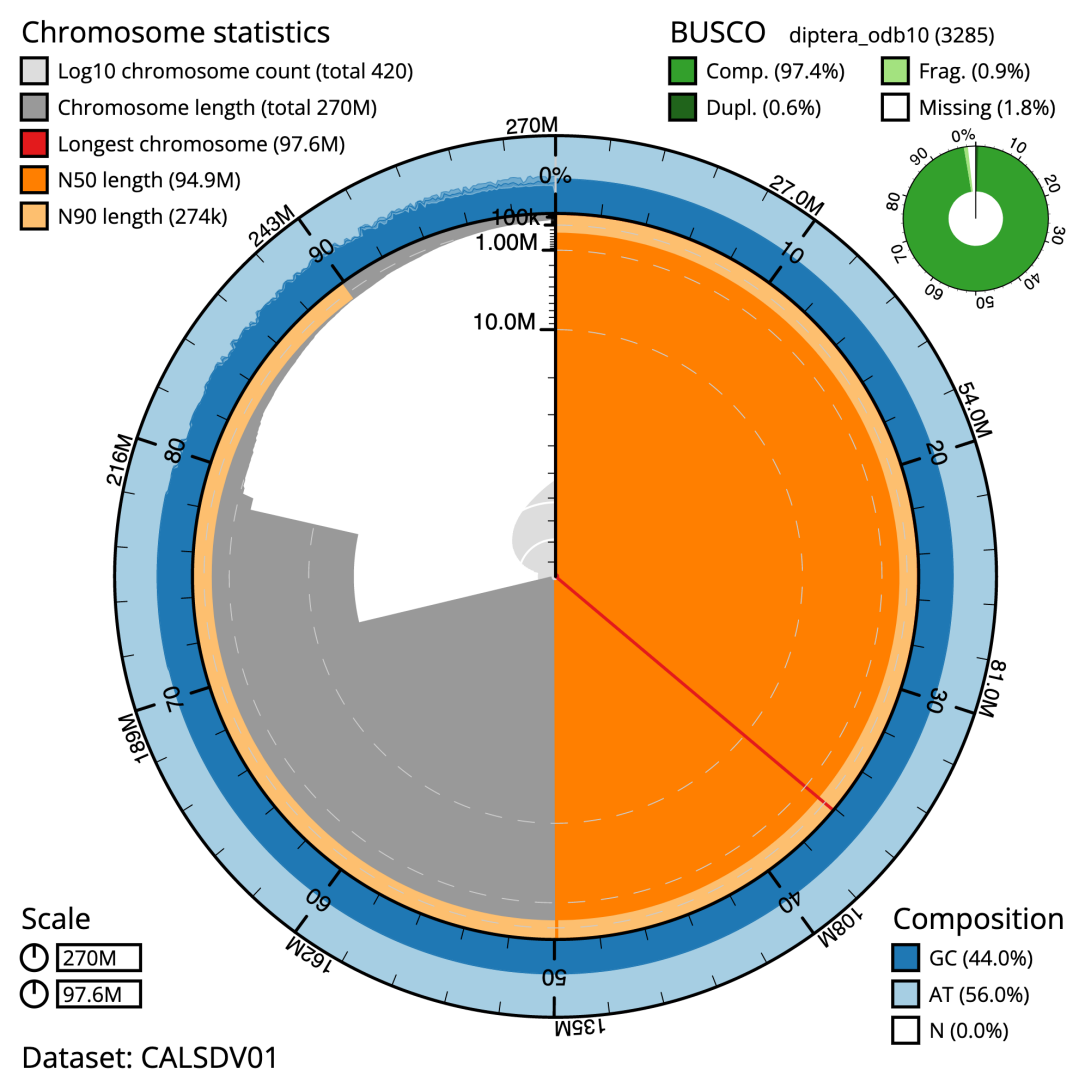
Snail plot summary of assembly statistics for *Anopheles coustani* assembly idAnoCousDA_361_x.2. The main plot is divided into 1,000 size-ordered bins around the circumference with each bin representing 0.1% of the 269,999,061 bp assembly. The distribution of sequence lengths is shown in dark grey with the plot radius scaled to the longest sequence present in the assembly (97,602,170 bp, shown in red). Orange and pale-orange arcs show the N50 and N90 sequence lengths (94,852,749 and 274,232 bp), respectively. The pale grey spiral shows the cumulative sequence count on a log scale with white scale lines showing successive orders of magnitude. The blue and pale-blue area around the outside of the plot shows the distribution of GC, AT and N percentages in the same bins as the inner plot. A summary of complete, fragmented, duplicated and missing BUSCO genes in the diptera_odb10 set is shown in the top right. An interactive version of this figure is available at https://blobtoolkit.genomehubs.org/view/Anopheles%20coustani/dataset/CALSDV01/snail.

**Figure 2.**
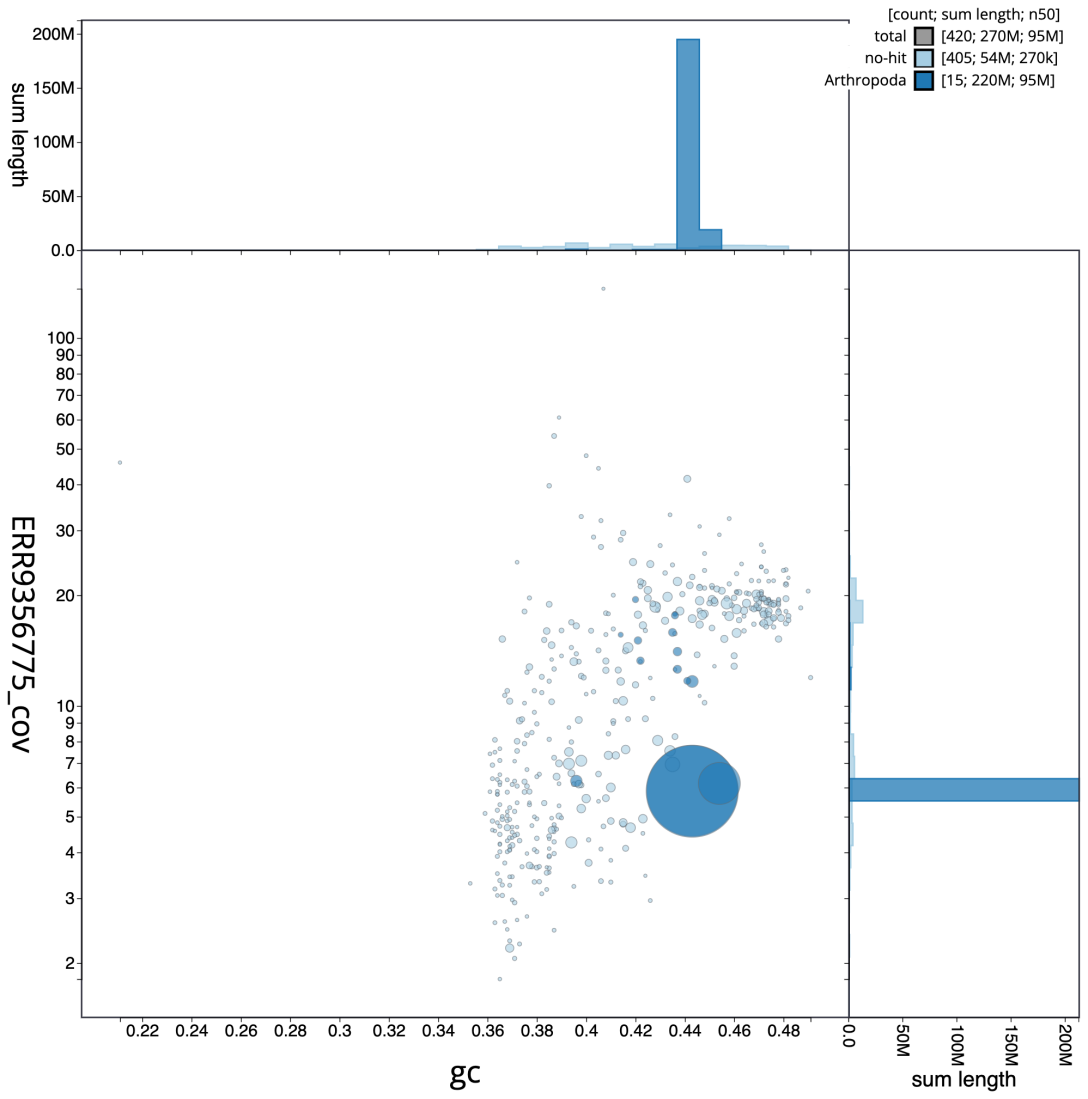
Blob plot of base coverage in a subset of idAnoCousDA_361_x 10x linked reads against GC proportion for *An. coustani* assembly idAnoCousDA_361_x.2. Chromosomes are coloured by phylum. Circles are sized in proportion to chromosome length. Histograms show the distribution of chromosome length sum along each axis. An interactive version of this figure is available at https://blobtoolkit.genomehubs.org/view/Anopheles%20coustani/dataset/CALSDV01/blob.

**Figure 3.**
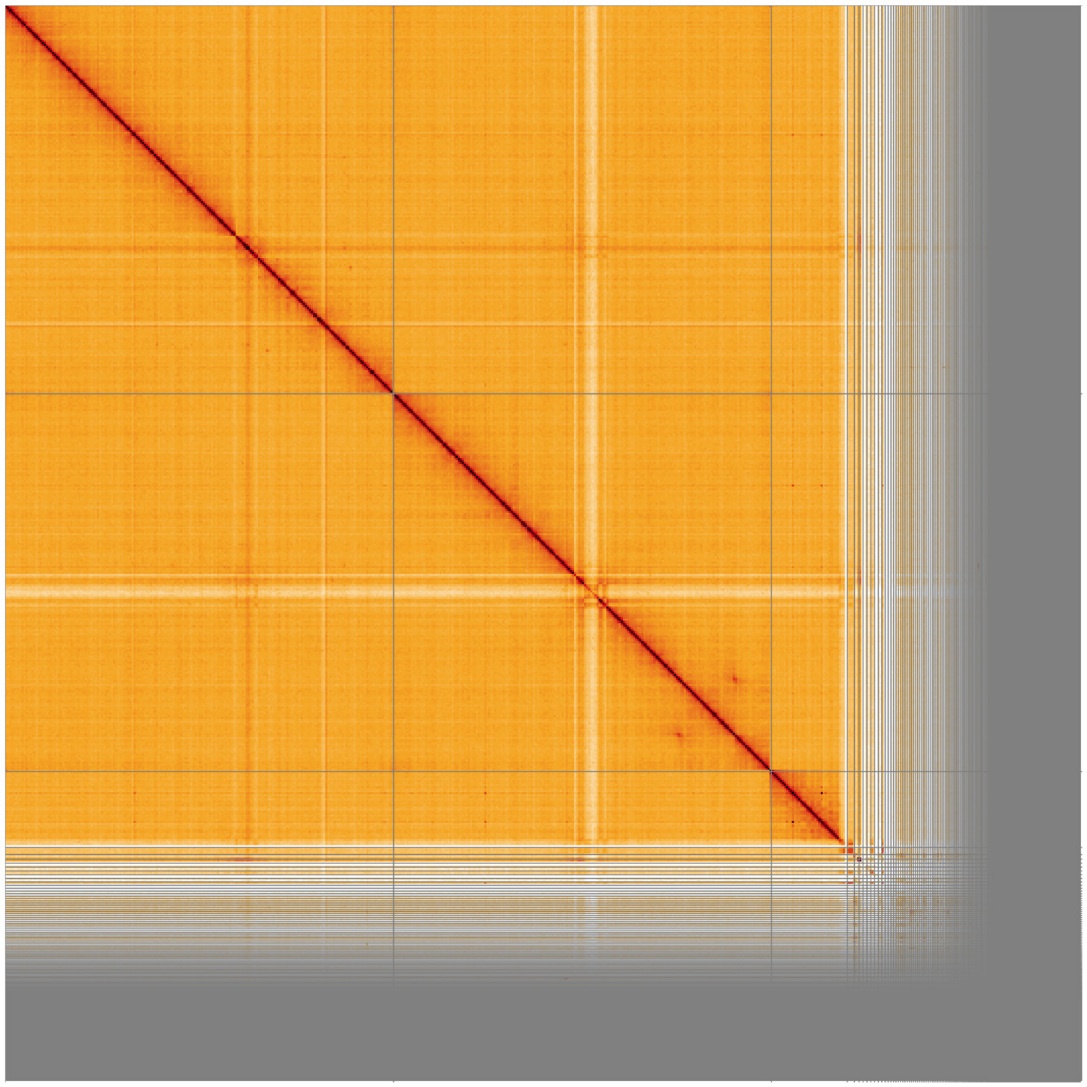
Genome assembly of *An. coustani*, idAnoCousDA_361_x.2: Hi-C contact map. Visualised in HiGlass. Chromosomes order: 3RL, 2RL, X, then remaining samples. Off-diagonal signal in 2L indicates a heterozygous inversion in the individual idAnoCousDA-364_x used for Hi-C. The interactive Hi-C map can be viewed at https://genome-note-higlass.tol.sanger.ac.uk/l/?d=TOv9LjXMTYKBy8dC3rTKgQ.

**Figure 4.**
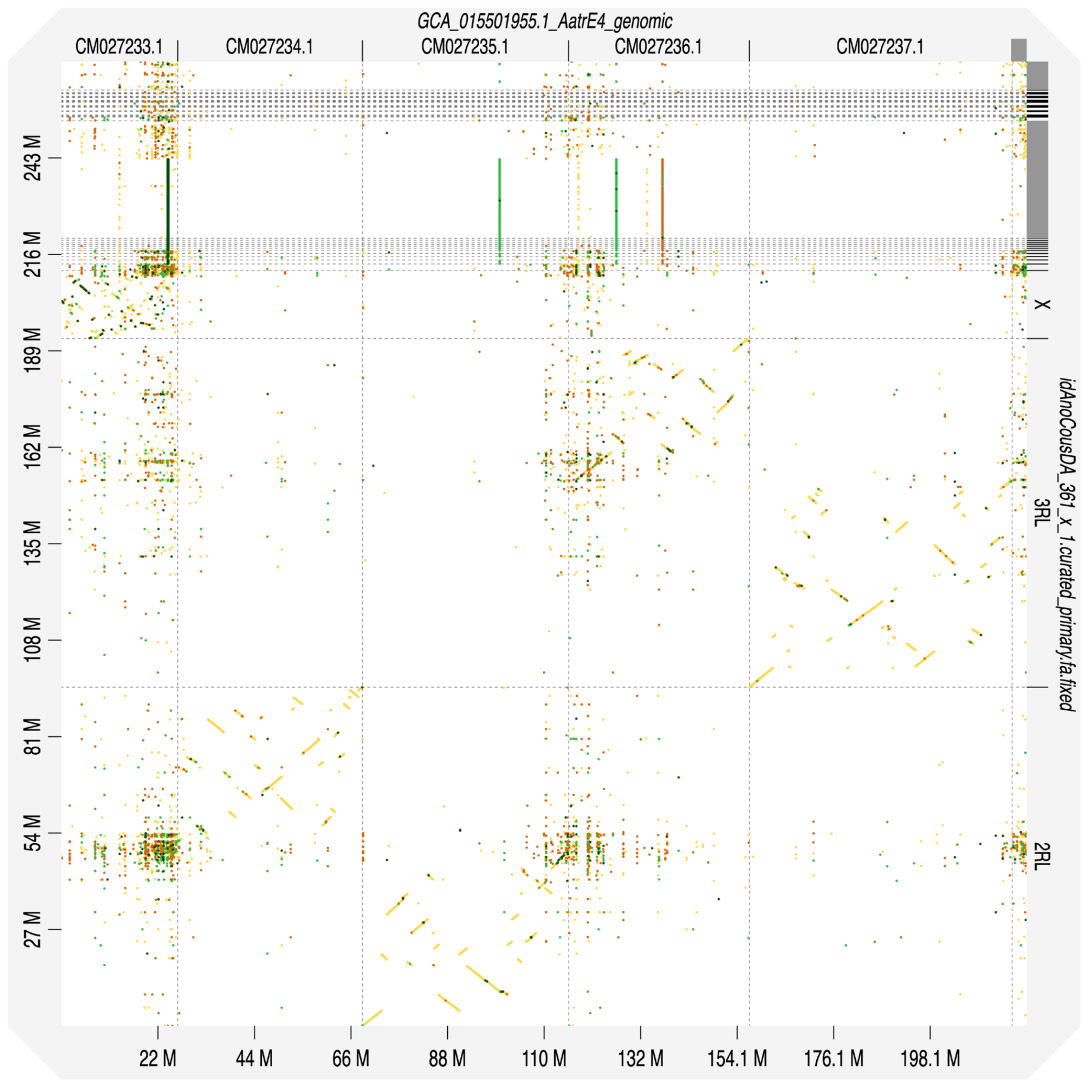
Alignment dotplot between genome assemblies of *An. coustani*, idAnoCousDA_361_x.2 and *An. atroparvus*, AatrE4. Visualised in D-Genies. Chromosome arms arrangement is the same for these representatives of *Anopheles* subgenus.

The final assembly has a total length of 270 Mb in 420 sequence scaffolds with a scaffold N50 of 94.852 Mb ( Figure 1– Figure 2; Table 1). The snail plot in Figure 1 provides a summary of the assembly statistics, while the distribution of assembly scaffolds on GC proportion and coverage is shown in Figure 2. 89.87% of the assembly sequence was assigned to three chromosomal-level scaffolds, representing two autosomes and the X sex chromosome ( Figure 3; Table 2). Chromosomes were numbered and oriented against the *An. atroparvus* assembly AatrE4 ^18^ (accession GCA_015501955.1) ( Figure 4) and double checked by polytene chromosome arms lengths, where 2L and 3R arms are the longest, 2R has intermediate length, followed by 3L and, finally, X ^14^. The assembled portion of chromosome 3RL is about 3Mbp longer than 2RL, which means the naming convention here of naming the longer chromosome as 2 is not precisely followed. The assembly has a BUSCO 5.3.2 ^19^ completeness of 97.4% using the diptera_odb10 reference set. While not fully phased, the assembly deposited is of one haplotype, and also includes the circular mitochondrial genome. Contigs corresponding to the second haplotype have also been deposited.

**Table 1.** Genome data for *An. coustani*, idAnoCousDA_361_x.

Project accession data	
Assembly identifier	idAnoCousDA_361_x.2
Species	*Anopheles coustani*
Specimen	idAnoCousDA-361_x
NCBI taxonomy ID	139045
BioProject	PRJEB53256
BioSample ID	ERS10527346
Isolate information	female, whole organism
*Raw data accessions*	
PacificBiosciences SEQUEL II	ERR9439496
10X Genomics Illumina	ERR9356773, ERR9356774, ERR9356775, ERR9356776
Hi-C Illumina	ERR9356772
PolyA RNA-Seq Illumina	ERR9356777, ERR9356778
*Genome assembly*	
Assembly accession	GCA_943734705
Accession of alternate haplotype	GCA_943734715
Span (Mb)	269.999
Number of contigs	448
Contig N50 length (Mb)	27.992
Number of scaffolds	420
Scaffold N50 length (Mb)	94.852
Longest scaffold (Mb)	97.602
BUSCO * genome score	C:97.4%[S:96.3%,D:1.1%], F:0.8%,M:1.8%,n:3,285

* BUSCO scores based on the diptera_odb10 BUSCO set using BUSCO 5.3.2. C=complete [S=single copy, D=duplicated], F=fragmented, M=missing, n=number of orthologues in comparison. A full set of BUSCO scores is available at https://blobtoolkit.genomehubs.org/view/Anopheles%20coustani/dataset/CALSDV01/busco.

**Table 2.** Chromosomal pseudomolecules in the genome assembly of *An. coustani*, idAnoCousDA_361_x.2.

INSDC accession	Chromosome	Size (Mb)	Count	Gaps
OX030900.2	2RL	94.853	1	3
OX030901.1	3RL	97.602	1	5
OX030902.1	X	19.034	1	4
OX030903.1	MT	0.015	1	0
	X Unlocalised	31.162	166	3
	Unplaced	27.333	250	13

**Figure 5.**
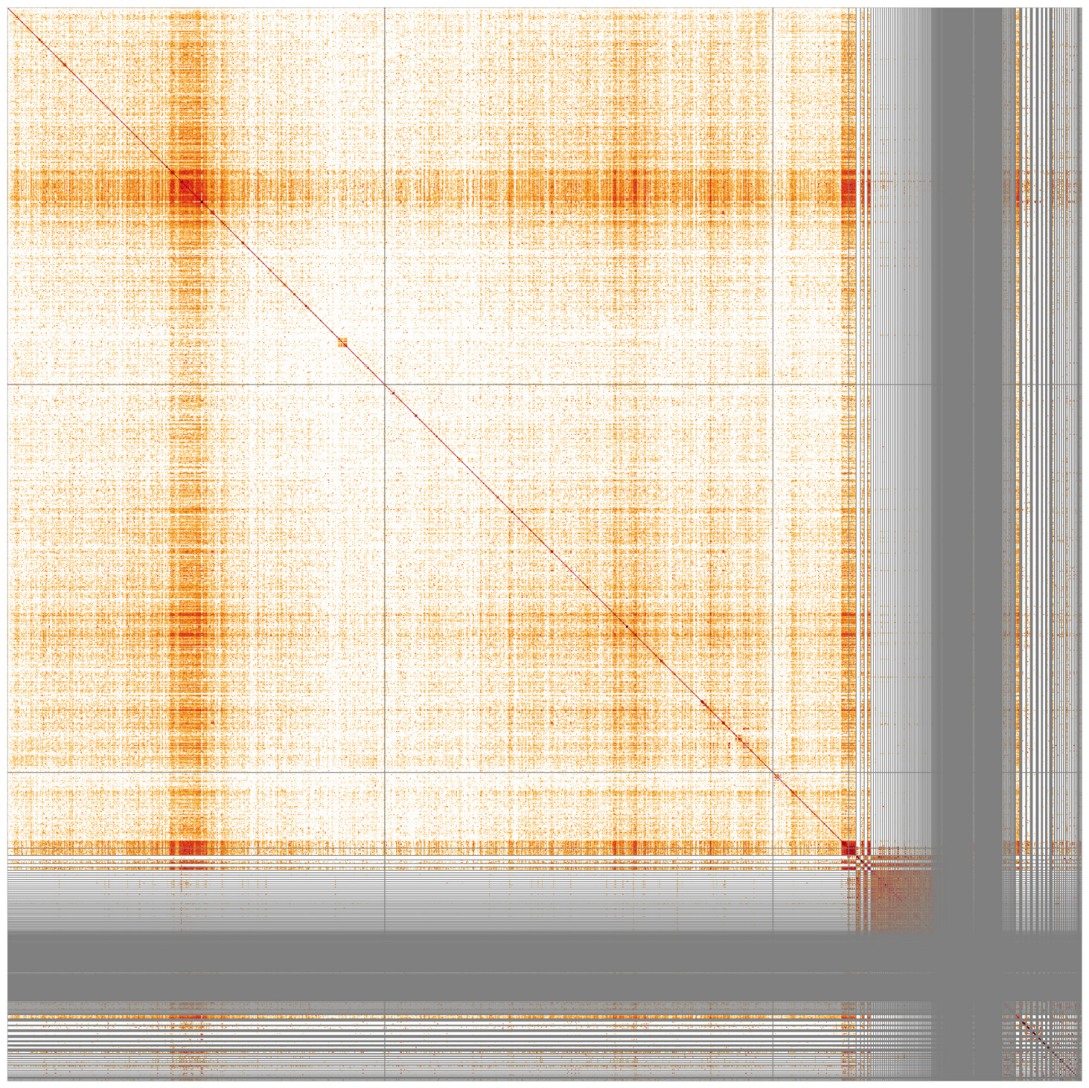
Sequence similarity heatmap for genome assembly of *An. coustani*, idAnoCousDA_361_x.2. Produced with StainedGlass, visualised in HiGlass. Chromosomes order: 2RL, 3RL, X - followed by the remaining scaffolds. Darker colours represent higher sequence similarity, notably at pericentric and intercalary heterochromatin as well as in unassembled X-linked scaffolds.

Chromosome arms, candidate centromere sequences, and the rDNA regions were delineated based on the presence of characteristic tandem repeat arrays ( Figure 5; Table 3). Candidate centromere regions of autosomes 2RL and 3RL comprised 52-53bp tandem repeat blocks with questionable sequence homology between chromosomes. On 3RL, a more pronounced tandem repeat region was found. Predicted centromere locations agree well with Hi-C signal ( Figure 3) and synteny to *An. atroparvus* ( Figure 4). In X chromosome assembly, no plausible centromere region was found. rDNA clusters were scattered across unlocalised X-linked scaffolds; they were often associated with tandem repeat blocks with unit length of 737 bp.

**Table 3.** Chromosome arms in the genome assembly of *An. coustani*, idAnoCousDA_361_x.2.

Chromosome	Start	End	Chromosome arm
2RL	1	48,615,516	2R
2RL	49,081,485	94,852,749	2L
3RL	1	57,704,850	3R
3RL	57,761,701	97,602,170	3L
X	1	19,033,788	X

Gene annotation was performed with NCBI Eukaryotic Genome Annotation Pipeline and is available in the RefSeq ^20^ under the accession GCF_943734705.1. A total of 14,493 genes were predicted, including 12,032 protein-coding genes and 2,426 non-coding RNAs. The genome assembly and gene annotations are hosted on VectorBase, www.vectorbase.org ^21^ under the identifier AcouGA1.

## Methods

### Sample acquisition and nucleic acid extraction

*Anopheles coustani* female individuals were caught in Lopé, Gabon using an animal-bait catch ^22^. A single female idAnoCousDA-361_x was used for Pacific BioSciences and 10x genomics, an unrelated female idAnoCousDA-364_x was used for Arima Hi-C.

For high molecular weight (HMW) DNA extraction one whole insect (idAnoCousDA-361_x) was disrupted by manual grinding with a blue plastic pestle in Qiagen MagAttract lysis buffer and then extracted using the Qiagen MagAttract HMW DNA extraction kit with two minor modifications ^23^. The quality of the DNA was evaluated using an Agilent FemtoPulse to ensure that most DNA molecules were larger than 30 kb, and preferably > 100 kb. In general, single mosquito extractions ranged in total estimated DNA yield from 200 ng to 800 ng, with an average yield of 500 ng. Low molecular weight DNA was removed using 0.8X AMpure XP purification. A small aliquot (less than ~5% of the total volume) of HMW DNA was set aside for 10X Linked Read sequencing and the rest of the DNA was sheared to an average fragment size of 12 to 20 kb using a Diagenode Megaruptor 3 at speeds ranging from 27 to 30. Sheared DNA was purified using AMPure PB beads with a 1.8X ratio of beads to sample to remove the shorter fragments and concentrate the DNA sample. The concentration and quality of the sheared and purified DNA was assessed using a Nanodrop spectrophotometer and Qubit Fluorometer with the Qubit dsDNA High Sensitivity Assay kit. Fragment size distribution was evaluated by running the sheared and cleaned sample on the FemtoPulse system once more. The median DNA fragment size for *Anopheles* mosquitoes was 15 kb and the median yield of sheared DNA was 200 ng, with samples typically losing about 50% of the original estimated DNA quantity through the process of shearing and purification.

For Hi-C data generation, a separate unrelated mosquito specimen (idAnoCousDA-364_x) was used as input material for the Arima V2 Kit according to the manufacturer’s instructions for animal tissue. This approach of using a sibling was taken to enable all material from a single specimen to contribute to the PacBio data generation given we were not always able to meet the minimum suggested guidance of starting with > 300 ng of HMW DNA from a specimen. Samples proceeded to the Illumina library prep stage even if they were suboptimal (too little tissue) going into the Arima reaction.

To assist with gene annotation, RNA was extracted from separate whole unrelated insect specimens (idAnoCousDA-54_x and idAnoCousDA-63_x) using TRIzol, according to the manufacturer’s instructions. RNA was then eluted in 50 μl RNAse-free water, and its concentration was assessed using a Nanodrop spectrophotometer and Qubit Fluorometer using the Qubit RNA Broad-Range (BR) Assay kit. Analysis of the integrity of the RNA was done using Agilent RNA 6000 Pico Kit and Eukaryotic Total RNA assay. Samples were not always ideally preserved for RNA, so qualities varied but all were sequenced anyway.

## Sequencing

We prepared libraries as per the PacBio procedure and checklist for SMRTbell Libraries using Express TPK 2.0 with low DNA input. Every library was barcoded to support multiplexing. Final library yields ranged from 20 ng to 100 ng, representing only about 25% of the input sheared DNA. Libraries from two specimens were typically multiplexed on a single 8M SMRT Cell. Sequencing complexes were made using Sequencing Primer v4 and DNA Polymerase v2.0. Sequencing was carried out on the Sequel II system with 24-hour run time and 2-hour pre-extension. A 10X Genomics Chromium read cloud sequencing library was also constructed according to the manufacturer’s instructions (this product is no longer available). Only 0.5 ng of DNA was used and only 25–50% of the gel emulsion was put forward for library prep due to the small genome size. For Hi-C data generation, following the Arima HiC V2 reaction, samples were processed through Library Preparation using a NEB Next Ultra II DNA Library Prep Kit and sequenced aiming for 100x depth. RNA libraries were created using the directional NEB Ultra II stranded kit. Sequencing was performed by the Scientific Operations core at the Wellcome Sanger Institute on Pacific Biosciences SEQUEL II (HiFi), Illumina NovaSeq 6000 (10X and Hi-C), or Illumina HiSeq 4000 (RNAseq) instruments.

## Genome assembly

Assembly was carried out with Hifiasm ^24^; haplotypic duplications were identified and removed with purge_dups ^25^. One round of polishing was performed by aligning 10X Genomics read data to the assembly with LongRanger align, calling variants with FreeNayes ^26^. The assembly was then scaffolded with Hi-C data ^27^ using SALSA2 ^28^. The assembly was checked for contamination as described previously ^29^. Manual curation was performed using gEVAL ^30^, HiGlass ^31^ and Pretext ^32^. The mitochondrial genome was assembled using MitoHiFi ^33^, which performs annotation using MitoFinder ^34^. The genome was analysed and BUSCO scores were generated within the BlobToolKit environment ^35^. Synteny analysis was performed with D-GENIES ^36^. Repetitive sequences were visualised with StainedGlass ^37^ and tandem repeats were annotated with ULTRA ^38^. Table 4 contains a list of all software tool versions used, where appropriate.

**Table 4.** Software tools used.

Software tool	Version	Source
hifiasm	0.14	24
purge_dups	1.2.3	25
SALSA2	2.2-4c80ac1	28
longranger align	2.2.2	39
freebayes	1.3.1	26
MitoHiFi	2	33
gEVAL	N/A	30
HiGlass	1.11.6	31
PretextView	0.1.x	32
BlobToolKit	3.4.0	35
BUSCO	5.3.2	19
D-GENIES	1.4	36
StainedGlass	0.5	37
ULTRA	1.0.0 beta	38

## Ethics/compliance issues

The genetic resources accessed and utilised under this project were done so in accordance with the UK ABS legislation (Nagoya Protocol (Compliance) (Amendment) (EU Exit) Regulations 2018 (SI 2018/1393)) and the national ABS legislation within the country of origin, where applicable.

## Data Availability

European Nucleotide Archive: *Anopheles coustani* genome assembly, idAnoCousDA_361_x.2. Accession number PRJEB53256; https://identifiers.org/bioproject/PRJEB53256. The genome sequence is released openly for reuse. The *Anopheles coustani* genome sequencing initiative is part of the Anopheles Reference Genomes project PRJEB51690. All raw sequence data and the assembly have been deposited in INSDC databases. Raw data and assembly accession identifiers are reported in Table 1.

## Author information

Members of Wellcome Sanger Institute Scientific Operations: Sequencing Operations are listed here: https://doi.org/10.5281/zenodo.12165051.

## References

[1] Gillies MT Coetzee M : A supplement to the anophelinae of Africa south of the Sahara (Afrotropical region).THE SOUTH AFRICAN INSTITUTE FOR MEDICAL RESEARCH,1987. Reference Source

[2] Laveran A : Sur un anopheles provenant de Madagascar. *C R Seances Soc Biol Fil.* 1900;57:109–110.

[3] Ndiath MO Eiglmeier K Olé Sangba ML : Composition and genetics of malaria vector populations in the Central African Republic. *Malar J.* 2016;15(1): 387. 10.1186/s12936-016-1431-2 27456078 PMC4960874

[4] Fornadel CM Norris LC Franco V : Unexpected anthropophily in the potential secondary malaria vectors *Anopheles coustani* s.l. and *Anopheles squamosus* in Macha, Zambia. *Vector Borne Zoonotic Dis.* 2011;11(8):1173–1179. 10.1089/vbz.2010.0082 21142969 PMC3151625

[5] Finney M McKenzie BA Rabaovola B : Widespread zoophagy and detection of *Plasmodium* spp. in *Anopheles* mosquitoes in southeastern Madagascar. *Malar J.* 2021;20(1): 25. 10.1186/s12936-020-03539-4 33413398 PMC7791646

[6] Duchemin JB Tsy JM Rabarison P : Zoophily of *Anopheles arabiensis* and *An. gambiae* in Madagascar demonstrated by Odour-Baited Entry Traps. *Med Vet Entomol.* 2001;15(1):50–57. 10.1046/j.1365-2915.2001.00276.x 11297101

[7] Makanga B Costantini C Rahola N : “Show me which parasites you carry and I will tell you what you eat”, or how to infer the trophic behavior of hematophagous arthropods feeding on wildlife. *Ecol Evol.* 2017;7(19):7578–7584. 10.1002/ece3.2769 29043015 PMC5632637

[8] Goupeyou-Youmsi J Rakotondranaivo T Puchot N : Differential contribution of *Anopheles coustani* and *Anopheles arabiensis* to the transmission of *Plasmodium falciparum* and *Plasmodium vivax* in two neighbouring villages of Madagascar. *Parasit Vectors.* 2020;13(1): 430. 10.1186/s13071-020-04282-0 32843082 PMC7447585

[9] Nepomichene TNJJ Tata E Boyer S : Malaria case in Madagascar, probable implication of a new vector, *Anopheles coustani*. *Malar J.* 2015;14: 475. 10.1186/s12936-015-1004-9 26620552 PMC4666205

[10] Antonio-Nkondjio C Kerah CH Simard F : Complexity of the malaria vectorial system in cameroon: contribution of secondary vectors to malaria transmission. *J Med Entomol.* 2006;43(6):1215–1221. 10.1603/0022-2585(2006)43[1215:cotmvs]2.0.co;2 17162956

[11] Nepomichene TNJJ Raharimalala FN Andriamandimby SF : Vector competence of *Culex antennatus* and *Anopheles coustani* mosquitoes for Rift Valley Fever Virus in Madagascar. *Med Vet Entomol.* 2018;32(2):259–262. 10.1111/mve.12291 29383746

[12] Diallo D Sall AA Diagne CT : Zika virus emergence in mosquitoes in southeastern Senegal, 2011. *PLoS One.* 2014;9(10): e109442. 10.1371/journal.pone.0109442 25310102 PMC4195678

[13] Boundenga L Makanga B Ollomo B : Haemosporidian parasites of antelopes and other vertebrates from Gabon, Central Africa. *PLoS One.* 2016;11(2): e0148958. 10.1371/journal.pone.0148958 26863304 PMC4749209

[14] Coetzee M : Chromosomal and cross-mating evidence for two species within *Anopheles (A.) coustani* (diptera: culicidae). *Syst Entomol.* 1983;8(2):137–141. 10.1111/j.1365-3113.1983.tb00473.x

[15] Ciubotariu II Jones CM Kobayashi T : Genetic diversity of *Anopheles coustani* (diptera: culicidae) in malaria transmission foci in Southern and Central Africa. *J Med Entomol.* 2020;57(6):1782–1792. 10.1093/jme/tjaa132 32614047 PMC7899271

[16] Campos M Crepeau M Lee Y : Complete mitogenome sequence of *Anopheles coustani* from São Tomé Island. *Mitochondrial DNA B Resour.* 2020;5(3):3376–3378. 10.1080/23802359.2020.1823273 33458175 PMC7782027

[17] Huho BJ Ng’habi KR Killeen GF : Nature beats nurture: a case study of the physiological fitness of free-living and laboratory-reared male *Anopheles gambiae s.l.* *J Exp Biol.* 2007;210(Pt 16):2939–2947. 10.1242/jeb.005033 17690243

[18] Lukyanchikova V Nuriddinov M Belokopytova P : *Anopheles* mosquitoes reveal new principles of 3D genome organization in insects. *Nat Commun.* 2022;13(1): 1960. 10.1038/s41467-022-29599-5 35413948 PMC9005712

[19] Simão FA Waterhouse RM Ioannidis P : BUSCO: assessing genome assembly and annotation completeness with single-copy orthologs. *Bioinformatics.* 2015;31(19):3210–3212. 10.1093/bioinformatics/btv351 26059717

[20] Pruitt KD Brown GR Hiatt SM : RefSeq: An update on mammalian reference sequences. *Nucleic Acids Res.* 2014;42(Database issue):D756–63. 10.1093/nar/gkt1114 24259432 PMC3965018

[21] Giraldo-Calderón GI Harb OS Kelly SA : VectorBase.org updates: bioinformatic resources for invertebrate vectors of human pathogens and related organisms. *Curr Opin Insect Sci.* 2022;50: 100860. 10.1016/j.cois.2021.11.008 34864248 PMC9133010

[22] Service MW : Mosquito ecology: field sampling methods. 2nd ed. Springer Netherlands,1993. 10.1007/978-94-011-1868-2

[23] Teltscher F Johnson H Lawniczak M : Manual extraction of High Molecular Weight DNA from single mosquitoes using the Qiagen MagAttract HMW DNA kit.2023; [cited 8 Jan 2024]. 10.17504/protocols.io.n92ldp6ool5b/v1

[24] Cheng H Concepcion GT Feng X : Haplotype-resolved *de novo* assembly using phased assembly graphs with hifiasm. *Nat Methods.* 2021;18(2):170–175. 10.1038/s41592-020-01056-5 33526886 PMC7961889

[25] Guan D McCarthy SA Wood J : Identifying and removing haplotypic duplication in primary genome assemblies. *Bioinformatics.* 2020;36(9):2896–2898. 10.1093/bioinformatics/btaa025 31971576 PMC7203741

[26] Garrison E Marth G : Haplotype-based variant detection from short-read sequencing. *arXiv [q-bio.GN].* 2012. 10.48550/arXiv.1207.3907

[27] Rao SSP Huntley MH Durand NC : A 3D map of the human genome at kilobase resolution reveals principles of chromatin looping. *Cell.* 2014;159(7):1665–1680. 10.1016/j.cell.2014.11.021 25497547 PMC5635824

[28] Ghurye J Rhie A Walenz BP : Integrating Hi-C links with assembly graphs for chromosome-scale assembly. *PLoS Comput Biol.* 2019;15(8): e1007273. 10.1371/journal.pcbi.1007273 31433799 PMC6719893

[29] Howe K Chow W Collins J : Significantly improving the quality of genome assemblies through curation. *GigaScience.* 2021;10(1): giaa153. 10.1093/gigascience/giaa153 33420778 PMC7794651

[30] Chow W Brugger K Caccamo M : gEVAL - a web-based browser for evaluating genome assemblies. *Bioinformatics.* 2016;32(16):2508–2510. 10.1093/bioinformatics/btw159 27153597 PMC4978925

[31] Kerpedjiev P Abdennur N Lekschas F : HiGlass: web-based visual exploration and analysis of genome interaction maps. *Genome Biol.* 2018;19(1): 125. 10.1186/s13059-018-1486-1 30143029 PMC6109259

[32] PretextView: OpenGL powered pretext contact map viewer.Github. Reference Source

[33] Uliano-Silva M Ferreira JGRN Krasheninnikova K : MitoHiFi: a python pipeline for mitochondrial genome assembly from PacBio high fidelity reads. *BMC Bioinformatics.* 2023;24(1): 288. 10.1186/s12859-023-05385-y 37464285 PMC10354987

[34] Allio R Schomaker-Bastos A Romiguier J : MitoFinder: efficient automated large-scale extraction of mitogenomic data in target enrichment phylogenomics. *Mol Ecol Resour.* 2020;20(4):892–905. 10.1111/1755-0998.13160 32243090 PMC7497042

[35] Challis R Richards E Rajan J : BlobToolKit – interactive quality assessment of genome assemblies. *G3 (Bethesda).* 2020;10(4):1361–1374. 10.1534/g3.119.400908 32071071 PMC7144090

[36] Cabanettes F Klopp C : D-GENIES: dot plot large genomes in an interactive, efficient and simple way. *PeerJ.* 2018;6: e4958. 10.7717/peerj.4958 29888139 PMC5991294

[37] Vollger MR Kerpedjiev P Phillippy AM : StainedGlass: interactive visualization of massive tandem repeat structures with identity heatmaps. *Bioinformatics.* 2022;38(7):2049–2051. 10.1093/bioinformatics/btac018 35020798 PMC8963321

[38] Olson D Wheeler T : ULTRA: a model based tool to detect tandem repeats. *ACM BCB.* 2018;2018:37–46. 10.1145/3233547.3233604 31080962 PMC6508075

[39] Long ranger BASIC and ALIGN pipelines. Software -Genome & Exome -Official 10x Genomics Support, [cited 16 Dec 2022]. Reference Source

