# Supplementary material for: Chromosomal reference genome sequences for the malaria mosquito, Anopheles coustani, Laveran, 1900
Source: Wellcome Open Res. 2024 Sep 26;9:551. [Version 1] doi: 10.12688/wellcomeopenres.22983.1 (PMC11490835; doi:10.12688/wellcomeopenres.22983.1)
Supplement: Peer review history

# Reviewer report: Reviewer response for version 1

**DOI:** 10.21956/wellcomeopenres.25308.r103180

**Published:** 2024-10-19

**Authors:** Daibin Zhong

**Affiliations:** 1 University of California, California, USA

The study by Bouafou et al., titled "Chromosomal Reference Genome Sequences for the Malaria Mosquito, *Anopheles coustani*, Laveran, 1900," presents a genome assembly from a single wild-caught female *An. coustani* collected in Lopé, Gabon. The mosquito's DNA and RNA were sequenced using Pacific Biosciences and Illumina technologies (10X Genomics, Hi-C, and RNAseq). Chromosome conformation Hi-C data from an unrelated female were utilized to scaffold the primary assembly contigs. The resulting genome sequence spans 270 megabases, with most of the assembly organized into three chromosomal pseudomolecules, and also includes the complete mitochondrial genome. The paper is clearly written, with a solid experimental design and appropriate statistical methods for genome assembly. It provides important new insights into the reference genome of this species, which will serve as a valuable resource for further research into the genetics and biology of *Anopheles coustani*, supporting the development of effective malaria control strategies. I have no further comments.

Are sufficient details of methods and materials provided to allow replication by others?

Yes

Is the rationale for creating the dataset(s) clearly described?

Yes

Are the datasets clearly presented in a useable and accessible format?

Yes

Are the protocols appropriate and is the work technically sound?

Yes

Reviewer Expertise:

population genomics

I confirm that I have read this submission and believe that I have an appropriate level of expertise to confirm that it is of an acceptable scientific standard.

# Reviewer report: Reviewer response for version 1

**DOI:** 10.21956/wellcomeopenres.25308.r103182

**Published:** 2024-10-17

**Authors:** Qian Han

**Affiliations:** 1 Hainan University, Haikou, Hainan, China

The report titled “Chromosomal reference genome sequences for the malaria mosquito, *Anopheles* *coustani*, Laveran, 1900” authored by Lemonde B. A. Bouafou et al., presents a genome assembly of the *An. coustani* mosquito, a species known to transmit malaria. The study is significant as it aims to understand the genetic makeup and evolutionary history of this mosquito, which may be useful for developing effective control strategies against malaria.

 In this report, the team successfully generated a genome assembly using Pacific Biosciences SEQUEL II and Illumina sequencing technologies. The genome assembly statistics are provided, including the chromosome sizes and counts, and the BUSCO scores, which assess the completeness of the assembly. The article also includes a list of references, providing additional context and supporting information for the study.

 Overall, this report contributes valuable genomic data for *An. coustani*, which could aid in the development of targeted interventions to control malaria transmission. The comprehensive approach taken by the authors, including the use of multiple sequencing technologies and bioinformatics tools, ensures the reliability and accuracy of the genome assembly.

**Minor:**

 I would like to know some interesting aspects, but not sure if they could be done further in this report or in somewhere else. For example,

 (1) The similarities and differences between the genomes of *An. coustani *and other known major malaria vectors, such as *An. gambiae*. Do these differences explain the differences in their ability to transmit malaria?  

 (2) Are there any specific genes found in this genome that are involved in *Plasmodium *infection or transmission compared with other mosquito species?

 (3) The article mentions that the feeding preference of this species varies from animal to human preference, are there genes or regulatory regions in the genome that are associated with this behavioral plasticity?

Are sufficient details of methods and materials provided to allow replication by others?

Yes

Is the rationale for creating the dataset(s) clearly described?

Yes

Are the datasets clearly presented in a useable and accessible format?

Yes

Are the protocols appropriate and is the work technically sound?

Yes

Reviewer Expertise:

vector biology, vector borne diseases, parasitology, structural biology.

I confirm that I have read this submission and believe that I have an appropriate level of expertise to confirm that it is of an acceptable scientific standard.
